# Factors Related to Breastfeeding Support in Lebanese Daycare Centers: A Qualitative Study among Daycare Directors and Employees

**DOI:** 10.3390/ijerph18126205

**Published:** 2021-06-08

**Authors:** Maya Abou Jaoude, Sara Moukarzel, Stef P. J. Kremers, Jessica S. Gubbels

**Affiliations:** 1 Department of Health Promotion, NUTRIM School of Nutrition and Translational Research in Metabolism, Maastricht University Medical Centre, P.O. Box 616, 6200 MD Maastricht, The Netherlands; s.kremers@maastrichtuniversity.nl (S.P.J.K.); jessica.gubbels@maastrichtuniversity.nl (J.S.G.); 2 Faculty of Nursing and Health Sciences, Notre Dame University, P.O. Box 72, Zouk Mikael, Lebanon; 3 Larsson-Rosenquist Foundation Mother-Milk-Infant Center of Research Excellence, University of California San Diego, La Jolla, CA 92161, USA; smoukarzel@ucsd.edu

**Keywords:** breastfeeding, daycare, supporting factors, barriers, cognitive determinants, environment, qualitative, Lebanon, policy

## Abstract

Breastfeeding has an important impact on maternal and child health, and is affected by several factors influencing its initiation and continuation. This qualitative study aimed to assess the main promoting and hindering factors for breastfeeding support in Lebanese daycare centers (DCCs), through the perspective of their directors and employees. The study was based on semi-structured interviews with 13 directors and 9 employees. It explored the influence of various cognitive factors as well as different environment types (physical, economic, political, and sociocultural) on their breastfeeding support. Findings suggested the directors and employees valued improving breastfeeding support in DCCs and the physical set-up of the DCCs allowed for this. However, various other factors restricted their abilities to support breastfeeding in the DCC, including limited knowledge and training on the topic, absence of written internal policies on breastfeeding, lack of enforcement in the application of national policies, and the current mothers’ work policies that negatively influenced the decision to breastfeed. Improvements could be achieved through integrated interventions, targeting the interaction of internal DCCs factors and national and DCCs breastfeeding policies, as well as several social factors, to create a multilevel approach targeting breastfeeding continuation support in breastfeeding-friendly DCCs in Lebanon and the region.

## 1. Introduction

Promoting and supporting breastfeeding is a well-known effective public health intervention associated with reduced mortality and morbidity among infants and mothers. Breastfeeding promotes optimal infant growth, cognitive development and immune function, as well as reducing the risk of maternal breast cancer and cardio metabolic diseases [1,2]. The World Health Organization (WHO) recommends infants to be exclusively breastfed for the first 6 months of life and favors continuation in combination with complementary feeding of solid foods for 2 years and beyond [3]. Despite these well-disseminated recommendations, breastfeeding rates remain suboptimal in many countries, including those in the Middle East–North Africa region such as Lebanon [2,4,5,6,7], where approximately only 38% of infants are exclusively breastfed during the first month of life and around 2% at 6 months of age [8,9]. In 2016, 16.5% of mothers of toddlers enrolled in daycare centers breastfed exclusively for 4 to 6 months and the mean age of formula introduction was around 2 months [6]. Thus, there is a clear need to improve breastfeeding rates in order to comply with the WHO Global Strategy for Women’s, Children’s and Adolescents’ Health (2016–2030) and promote infants’, children’s, and women’s health [10].

Various endeavors to help with initiation and continuation of breastfeeding have been implemented globally. A top-down approach mainly aims at improving and implementing national breastfeeding policies and programs [11,12,13], whereas the bottom-up approach relies on assessing and attending to individual-level barriers to breastfeeding initiation and sustenance, thereby affecting the community’s breastfeeding support [14,15,16,17]. Consequential approaches include multifaceted breastfeeding support in society (e.g., through daycare centers, baby-friendly hospitals, workplace policies) with the help of healthcare providers (such as nurses, lactation consultants and daycare center employees), while targeting the family’s supportive role [8,18,19,20,21,22,23,24,25].

Daycare centers (DCCs) play a crucial role in the social environment of breastfeeding mothers, contributing to the lengthening and continued support of breastfeeding [26,27,28]. Indeed, it is the setting where children of working mothers spend most of their early years after maternity leave ends. In Lebanon, at least 25% of parents rely on daycare center support [29]. Lundquist et al. (2019) highlighted educating DCCs providers about breastfeeding as well as creating supportive policies in DCCs as promising breastfeeding promotion strategies [27]. Furthermore, Marhefka et al. (2019) concluded that breastfeeding-friendly childcare centers could contribute to creating a cultural shift towards breastfeeding continuation [28]. However, Mattar et al. (2019) showed very low rates of breastfeeding among children in Lebanese daycare centers. This shows the urgent need for breastfeeding initiation and continuation support, highlighting its importance when considering the elevated risk of recurrent illnesses in children attending daycare centers [6].

In view of this worrying situation, determining the supports and constraints for effective breastfeeding support practices in DCCs is a critical step that could assist in designing and implementing targeted interventions and policies aimed at improving breastfeeding support, not only in Lebanese daycare centers but also in other countries inside and outside the Middle Eastern region. In a country where healthcare resources are restricted [30], addressing breastfeeding support will benefit the society as a whole, and contribute to optimizing young children’s health in Lebanon. The purpose of this study is therefore to perform an in-depth qualitative assessment of the factors hindering and promoting breastfeeding support in Lebanese daycare centers, based on the perspectives of daycare directors and employees. This assessment will give insight into the present situation regarding breastfeeding support in Lebanese daycare centers and provide indications for possible future interventions.

## 2. Materials and Methods

This study is a qualitative research study, based on interviews with a purposive sample of daycare center directors and employees working in the region of Mount Lebanon, a peri-urban region in the country of Lebanon. It examines the different barriers and supports regarding breastfeeding in daycare centers based on the employees’ and directors’ perspectives.

### 2.1. Setting

In Lebanon, daycare centers are private establishments that provide supervision for infants and children up to 4 years of age during the daytime, usually from the end of maternity leave (around the child’s age of 3 months) or later. As per the requirements of the Ministry of Public Health (MOPH) [31], daycare centers have separate classes based on the children’s ages. At 4 years, children in Lebanon usually enroll in primary schools. Daycare centers are managed by directors and operated by a number of employees. Directors are generally responsible for the daycare center management (including first contact with parents) and overseeing care for children, as well as employee supervision. They are usually on the premises during opening hours, but they are not primary caregivers for the children. Daycare center employees can have a variety of educational backgrounds, ranging from childhood education or nursing to psychology or psychomotricity. They spend their day with the children, teaching them in the classrooms or performing specialized developmental activities with them. The employees are also usually responsible for feeding the child and milk handling. Parents pay tuition fees on a monthly basis in most daycare centers. These fees vary according to the daycare centers’ location and specific services provided (for example, speech therapists, child psychologists, and other skilled personnel providing activities). Food is usually provided for older children at all meal and snack times. Parents are responsible for providing powdered or breastmilk.

### 2.2. Participants and Procedures

Two types of participants were recruited for the study: daycare center directors and daycare center employees. The employees selected for this study were those supervising children aged less than 2.5 years, as the recommendations of the WHO/Lebanese Ministry of Public Health (MOPH) for breastfeeding applies up to at least two years [32,33].

The primary investigator (MAJ) invited directors from a purposive sample of 16 licensed DCCs located in socioeconomically diverse neighborhoods by phone to participate in the study. Since the data collection was conducted directly after a COVID-19 lockdown, the DCCs’ selection was affected by which DCCs were open as well as the DCCs directors’ agreement to a face-to-face interview. Accordingly, the DCCs were selected from the MOPH and syndicate of daycare centers website, as well as from the researchers’ own networks, until satiation occurred in the interviews. In total, 13 directors (81.1%) consented to participate, six of them agreeing to have their employees approached; this resulted in nine employees participating, one of whom had the dual role of nurse and daycare center manager (D8/E6). Written consent was obtained from each participant.

### 2.3. Data Collection

The interviews were conducted using a semi-structured interview guide, previously piloted in a preliminary study targeting the barriers and supports regarding breastfeeding in daycare centers in the northern region of Lebanon (unpublished findings of a cross-sectional study using a mixed method approach conducted in the northern region of Lebanon on 30 licensed daycare centers). The interview guide was not validated. The selected interview questions were based on the different methods used and findings from previous research concerning barriers and supports regarding breastfeeding in daycare centers [27,28,34,35,36,37]. The questions explored potential individual-, group-, or society-level factors as well as policies and environmental determinants of the daycare center’s director and employee support of breastfeeding found in the designed conceptual framework (Figure S1), which was based on the EnRG framework [38], as well as on the conceptual framework of factors affecting breastfeeding based on a socioecological model [39]. The framework interlinked the different-level factors together and showed their mutual influence on each other. The individual-level factors mainly included cognitive determinants; the environmental factors included the home and peer environments as well as the work environment. Another important environmental influence was the mother, in addition to the public policy environment- and society-level factors that influence each other and in turn the individual-level factors.

The questions were designed to collect in-depth qualitative data about attitudes, knowledge, and other sociocognitive factors, training, and practices of the director/employee, as well as policies and barriers/supports affecting breastfeeding within the different environmental determinants like the home/work environment, the daycare center’s physical set-up and dynamics, and the mothers’ influence (Tables S1 and S2). The participants were able to elaborate on their vision and opinions on breastfeeding in the daycare center set-up in Lebanon, as well as inform the researchers about current on-premise practices.

Interviews were conducted in Arabic (84.6%) or French (15.4%), depending on the preferences of the interviewees. The interview guide was translated for this by a native Arabic and fluent French bilingual researcher (MAJ). The researchers continued recruiting participants for the interviews until data saturation was reached and interviews provided no additional information. This occurred after interviews with 13 managers and 9 employees, from a total of 16 DCCs daycare centers that were initially contacted. Since the data collection was performed during the COVID-19 pandemic, and due to the economic and safety burdens present in Lebanon at the time of the data collection, the primary investigator was forced to meet the employees and directors simultaneously in eight daycare centers, except for one daycare center where the employees were interviewed separately from the director.

Interviews were audio-recorded and transcribed into English by the principal investigator (MAJ), prior to being securely stored and password-secured. Two of the translated interviews were independently back-translated to Arabic by a second researcher (SM) to ensure translation accuracy and quality. Differences with original audio recordings were checked and discussed by both researchers.

### 2.4. Data Analyses

The responses to the different questions during the interview were coded using MAXQDA 2020 (version 20.3.0, VERBI software, Berlin, Germany) and analyzed through thematic analysis based on the theoretical framework, starting with open coding. The principal investigator reviewed the responses to the questions and determined the themes, resulting in the coding trees (Figures S2 and S3). A selected number of responses were reviewed by a second researcher (SM) to determine inter-rater reliability. If consensus was not reached, a third researcher (JG) was consulted to finalize the themes.

## 3. Results

### 3.1. General Sample Characteristics

The interviews lasted on average 38 min (range: 25 to 52 min). All directors and employees were women, with the exception of one male director, ranging in age from 30 to 65 years (See Table 1).

The directors and employees had different backgrounds, listed in Table 1 and Table 2. Most of them were mothers themselves, some having also breastfed their own children. In one of the centers, a nurse was an employee as well as being the manager of the center and was considered as a director/employee (C8: E1/D1). The DCCs originally provided care for 30 to 190 children, but due to government-imposed COVID-19-related restrictions on childcare capacity, this number was substantially lower in various centers at the time of the data collection (range: 14–100) (see Table 2). There was a consistently small number of breastfed children across the DCCs, ranging from zero to a maximum of four, which mostly received pumped breastmilk and were not breastfed onsite. Furthermore, most of the DCCs accepted infants of the age of 2–3 months, except for four centers accepting infants only after 6 months of age.

### 3.2. Cognitive Determinants

Various cognitive determinants of breastfeeding support were mentioned, which are discussed in more detail below. Quotes reflecting the various themes mentioned are presented in Table 3.

#### 3.2.1. Intention to Facilitate Breastfeeding and Perceived Behavioral Control

Interviewed directors and employees clearly stated that they intended to provide adequate assistance and facilitation to breastfeeding mothers in the DCC and perceived themselves capable of this, given their knowledge, material, and experience. Although some directors claimed that they could potentially have an impact on parents’ attitudes and mothers’ willingness to breastfeed, most of them believed the mothers (mostly affected by external factors themselves, like work settings and distance from DCCs) are in fact the sole decision maker concerning breastfeeding continuation in DCCs. This fact discouraged some of them to put in more effort to support breastfeeding. 

#### 3.2.2. Knowledge and Experience

Their educational background affected both the directors’ and employees’ support of breastfeeding. While those with a nursing background were generally most supportive, the employees and directors with another educational background differed in their support, depending on many other factors like age, motherhood, traditions, and culture.

In terms of training, most participants followed those set by the government as a requirement for every DCC. The employees and directors both preferred to participate in diverse training courses (preferably certified) on the topic of education and child behavior in general, rather than breastfeeding specifically. Technology facilitated employees’ and directors’ interest in and access to diverse topics, including breastfeeding. It encouraged communication between personnel as well as sharing of new information in groups.

#### 3.2.3. Attitude

All the interviewed directors and employees had a positive attitude regarding breastfeeding in general, and in DCCs specifically. They believed it had a very important impact on the health of both mother and child, with the exception of two directors who prioritized prompt post-delivery return to work and mentioned the inability to track the quantity of milk given and the child’s satiety as a limitation of breastfeeding.

Despite their positive attitude regarding breastfeeding, most directors stated that they were not responsible for increasing the mothers’ awareness of its importance, claiming that it was not within their job description. In addition, some mentioned the on-premise presence of parents in general as being a burden, because they would be interfering with the daily practices in the DCC. This added to the current challenges in scheduling the mothers’ visits to the DCC and the employee shortage in the present economic situation in Lebanon.

The DCCs’ minimal age of acceptance for children also affected breastfeeding continuation support in the center. The DCCs that accepted younger infants showed the best encouragement and facilitation of breastfeeding. Some directors raised their concerns about the appropriate age at which to stop onsite breastfeeding for different reasons, including sexual labeling, leaving the baby on the breast all the time, and child–mother separation issues.

#### 3.2.4. Subjective Norm

Communication (preferably oral) between the director and the mother/parents appeared to be an effective booster for breastfeeding encouragement in the different DCCs. All directors stated that they had a definite impact on their employees’ encouragement of breastfeeding and that employees influenced each other positively in this regard. Furthermore, employees confirmed this and regarded the director as a role model.

### 3.3. Environmental Determinants

#### 3.3.1. Physical Environment

All interviewed directors and employees were well aware that a breastfeeding-friendly physical set-up should be private and comfortable in the DCC. All DCCs were very well equipped with the required appliances, like microwaves, fridges, and freezers. Furthermore, the directors stated that they always succeeded in having a private room (sometimes their own office), ready for the breastfeeding mother, not necessitating any extra cost.

#### 3.3.2. Economic Environment

Most directors indicated that facilitating breastfeeding continuation did not burden the employees with more work nor increase costs. Some indicated that this was due to the small number of mothers that actually breastfed. The DCCs’ management orientation also affected breastfeeding support: in more business-oriented DCCs, breastfeeding continuation was not of prime importance, but rather practicality. Employee turnover did not affect the continuation of breastfeeding support practices in the DCCs.

#### 3.3.3. Political Environment

The directors stated that employees were not aware of the details of national breastfeeding policies; they just knew that the mother had the right to breastfeed in the DCC. All directors agreed that gynecologists, pediatricians, nurses, and breastfeeding-friendly hospitals have an important role to play in breastfeeding support and suggested this should be addressed in national breastfeeding policies. They also suggested that the DCC syndicate should have a breastfeeding supportive role in a DCC and in implementing new guidelines that would oblige DCCs to accommodate and encourage breastfeeding mothers.

In the opinion of some directors, however, even if the government made breastfeeding support in DCCs obligatory, action first needed to be taken in women’s work settings, prior to changing policies in the DCC. The maternity leave duration was deemed too short for breastfeeding continuation and most work settings were not convenient for pumping breast milk. Some directors also stated that if one of their DCC employees was a mother, they would not approve of several breastfeeding breaks either. They claimed it would affect the work quality and professionalism of the employee.

Furthermore, some directors insisted that parents’ decisions and attitudes about breastfeeding were the only factor of importance, even if DCC policies were to change. All the directors regularly conducted a policy review, but few included any written policies on breastfeeding. Increased breastfeeding support was observed when it was included in the reviewed policy. Several DCC directors and employees discussed the lack of training offered by the government. They expressed the need for obligatory training programs on child pedagogy and breastfeeding to boost the interest of employees, directors, and even parents.

When asked about the possibility of having a breastfeeding-friendly DCC certification, all directors and employees were keen on applying it in their DCC, most also regardless of the cost or the changes required for certification. One director commented that it could create an added economic burden for DCCs, especially in the current situation and pandemic. Most participants stated that this certification would also encourage breastfeeding continuation and increase the perceived reliability of the DCC. Employees saw this certification as a boost for their career development and approved it directly. One director suggested having the Ministry of Public Health make this certification obligatory for DCCs. In her opinion, it would increase the enthusiasm to apply this certification in the DCC.

#### 3.3.4. Sociocultural Environment

All directors and employees stated that their practices were affected by their own social background, some more than others. On the other hand, they stated that mothers constituted one of the main pillars of their own sociocultural environment regarding breastfeeding. The DCC mother’s age, body image, generation, family, social background, education level, and nationality were identified as having an undeniable impact on her breastfeeding continuation in the DCC.

From the directors’ point of view, the employees’ background could have an impact on the support of breastfeeding in the DCC, although they believed personal decision and personality would be the most important determinants affecting employees’ breastfeeding support.

## 4. Discussion

This study aimed at assessing the determinants of breastfeeding support in Lebanese DCCs, from the viewpoints of directors and employees. Most of the study participants had a positive attitude regarding breastfeeding: they were willing to facilitate it for mothers and felt confident to do so. This was in line with studies from other countries, such as the USA [37] and New Zealand [40]. The mother was viewed as the most important influence in the sociocultural environment in our study: her decision to continue breastfeeding was the most important factor mentioned affecting directors’ and employees’ support. Similarly, Javanparast et al. (2012) found that the main role of DCC staff was to support the parental choice in terms of breastfeeding [41]. Furthermore, Lundquist et al. (2019) also mentioned that from the mothers’ point of view, DCCs’ directors and employees should support their decision regarding breastfeeding and not interfere with it [27].

Participants indicated that the mother herself was greatly influenced by several factors, like body image fears, physical difficulties, cultural influences, social background, age, and nationality. These factors are largely in line with barriers to breastfeeding in Lebanon in general [14,15], showing that misconceptions and stress, as well as physical difficulties, were among the factors hindering continuation of breastfeeding by Lebanese mothers. Mothers’ knowledge was also previously mentioned as a facilitator of breastfeeding continuation [14]. Our participants indicated that DCCs could play a positive role in promoting breastfeeding awareness and knowledge among mothers, in line with other studies [36,42].

Another important topic mentioned by the participants was the communication between employees and director, as well as among employees themselves, potentially reinforcing the staff’s breastfeeding support. The age at which DCCs accept children seemed to affect the level of staff support for breastfeeding continuation: the DCCs that accepted younger infants had the highest level of support, in line with findings from Mohd Suan et al. (2017). This indicates a need to focus on breastfeeding continuation support, beyond the initial three months, maximizing the chance of prolonging the breastfeeding period [36]. In line with this, Batan et al. (2013) showed a marked increase in the number of mothers breastfeeding up to 6 months as a result of higher breastfeeding support provision by the DCCs’ staff beyond the first few months [43].

Both the directors’ and employees’ educational background affected their support of breastfeeding in DCCs. Nurses seemed to be the most supportive, probably because breastfeeding is one of the main topics in their education. Participants with other educational backgrounds seemed to be more affected by their own social background, culture, and traditions rather than their education. In terms of training, most DCCs followed a list of training topics set by the government that did not include breastfeeding as a main topic, but focused on child safety and education. This discouraged DCC staff’s interest in the subject of breastfeeding, as seen in previous studies [35,36,44], indicating the need for improving initial and postgraduate DCCs’ directors’ and employees’ knowledge of breastfeeding as well as providing targeted government-organized training courses to enhance it and subsequently support it in DCCs. Furthermore, extending this training to the parents could also improve this support, as seen in other studies [36,37]. Our participants further indicated that technology could play a useful role here, being a major learning platform with easy access, as well as a means to share information among colleagues. This is in line with a study from the USA [34], where 88% of DCCs’ directors selected the internet (specifically a website specializing in breastfeeding) as a preferred means to obtain breastfeeding information that they can pass on to parents in DCCs. On the other hand, some of the directors in our study also mentioned the danger of getting non-scientific or incorrect information from the internet. This stresses the need for reliable online resources on breastfeeding.

When discussing the political environment, our study participants were aware of the National Breastfeeding Policy but were not familiar with its specific content and details. They only knew that there was a policy that permitted breastfeeding continuation in DCCs. This is consistent with the discrepancy between policy and its implementation by governmental institutions in Lebanon in general [45], as well as the fact that breastfeeding is not a priority at the national level [14]. Internally, most DCCs did not have specific written policies about breastfeeding. A study by Javanparast et al. (2012) in Australia stated that six out of 15 DCCs had a written breastfeeding policy with general guidelines, mainly about breastmilk handling and food safety procedures, but not breastfeeding support [41]. Co-creation of such policies with employees could improve their commitment as would the onsite knowledge of national policies. Interviewees also discussed the different challenges mothers faced, necessitating several workplace policies’ reconsideration, including lengthening of maternity leave and improvement of possible breastfeeding continuation conditions [15], to support efficient breastfeeding continuation in DCCs.

All DCCs in our sample had the necessary equipment for breastmilk handling and had no problem accommodating sufficient privacy and comfort for an incoming breastfeeding mother. However, they did not actively communicate this to the mothers. This highlights the importance of enforcing the application of the national breastfeeding policy in the DCCs, as was discussed in Mohd Suan et al. (2017) [36]. Indeed, orientation toward the practical application of breastfeeding policies enables the DCCs’ directors and employees to be confident in proposing breastfeeding continuation in DCCs and persuading mothers to practice it. Furthermore, initiation of breastfeeding certification for DCCs was discussed with the directors in the current study. Most of them agreed that it would be an encouraging factor for improving both the directors’ and employees’ attitude regarding breastfeeding support as well as the mothers’ will to continue breastfeeding in the DCC, in line with a previous study [36]. The additional cost and changes that certification would entail were accepted by most directors.

This research should be interpreted with several limitations in mind. Conducting the study during the COVID-19 pandemic led to several hindering factors in terms of data collection and interview responses. In addition, the pandemic might have also influenced the study findings: for instance, it resulted in extra costs (e.g., shoe covers, masks, sanitizer) and low practicality of breastfeeding due to hygiene, social distancing, and sanitation purposes in some but not all of the DCCs. The deteriorating economic situation in Lebanon as well as the obligatory reduction in the number of children attending the DCCs created stress for both directors and employees, modifying their responses accordingly. Furthermore, most directors who agreed to have their employees participate in our study requested to do the interview together with the latter to save time. This may have led to socially desirable answers by the employees. Additionally, some of the participants did not want to share their age or certain center characteristics in the interviews. The number of participating employees was further limited due to previously mentioned factors, although data saturation was reached. Although data satiation occurred for both directors and employees, the sample size was limited (*n* = 22). In addition, a purposive sample was used. In combination with the limitations the COVID-19 pandemic posed for the recruitment, this likely resulted in selection bias, potentially limiting generalizability of the findings. A quantitative study comprising a larger, representative sample can confirm our findings. In addition, although only one male director was included in the sample, this is reflective of the DCC practice in Lebanon in which male DCC directors are rare. One of the strengths of the study is that it is the first study to explore this subject in Lebanon. Furthermore, in several previous studies on the topic [34,35,40,41,46], the main research question centered around knowledge and attitude of daycare centers’ staff or policies supporting breastfeeding continuation in DCCs. Our study took a broader perspective, guided by an extensive theoretical framework, and included the interrelationships of relevant factors. 

## 5. Conclusions

Breastfeeding support in DCCs in Lebanon seems to be hindered by a wide variety of barriers that extend beyond individual-level factors, including sociocultural, economic, and political environmental factors. Improving breastfeeding support in DCCs was seen to be of key importance by the different DCC directors and employees. Our study showed that this should be done through changes in internal DCCs’ factors and breastfeeding policies, as well as sociocultural components (especially the mother) at many levels. Consequently, the development of a multilevel framework that identifies different variables associated with breastfeeding support in DCCs is needed to plan interventions that will result in creating a cultural shift toward improving breastfeeding support, continuation rates, access to breastfeeding-friendly DCCs, and therefore improving public health, not only in Lebanon but also in other countries around the world.

## Figures and Tables

**Table 1:** Sample characteristics.

DCC	Directors (*n* = 13) Employees *n* = 9)	Gender	Age	Education	Years in DCC	Motherhood	BF Experience
C1	D1	F	39	MA Psychotherapy	5	Y	Y
	E1	F	34	BS Education	3	Y	Y
	E2	F	32	BS Nursing	3	Y	Y
	E3	F	65	High school	3	Y	Y
C2	D2	F	33	BS Pedagogy MS Education	5	Y	Y
C3	D3	F	50	BS Childhood Education	27	Y	N
C4	D4	F	39	PhD Psychopathology and Analysis	9	N	N
C5	D5	F	43	BS Education	16	Y	Y
C6	D6	F	39	BS Education	14	Y	Y
	E4	F	35	BS Nursing	14	Y	Y
C7	D7	F	57	BS Education	6	Y	Y
	E5	F	34	BS Psychology	6	Y	Y
C8	D8/E6	F	33	BS Nursing MS Human Resources	13	N	N
C9	D9	F	32	BS Psychometry	5	Y	Y
C10	D10	F	37	BS Biology MS Industrial Technology	6	Y	Y
	E7	F	42	BS Nursing	5	Y	Y
C11	D11	M	30	BS Law	3	N	N
C12	D 12	F	NA	BS Nursing	20	Y	Y
	E8	F	45	BS Childhood Education	20	Y	N
	E9	F	NA	BS Childhood Education	12	Y	Y
C13	D13	F	31	BS Speech Therapy	4	Y	Y

Abbreviations: D = Director; E = Employee; C = Center; DCC = Daycare Center; BF = Breastfeeding; BS = Bachelor of Science; MS = Master of Science; Y = Yes; N = No; NA = Not Available.

**Table 2:** Daycare centers’ characteristics.

DCC	No. of Employees in DCC	Child Age Accepted in DCC	Total Children Pre-COVID-19	Current Number of Children in DCC	No. of Breastfed Children
C1	4	5 Months	55	15	2
C2	30	2 Months	150	<75	1
C3	12	2 Months	40	20	0
C4	12	10 Months	30	14	1
C5	37	2 Months	150	70	4
C6	25	2 Months	143	100	3
C7	NA	9 Months	90	45	2
C8	14	3 Months	70	23	3
C9	12	6 Months	85	0	3
C10	NA	3 Months	NA	NA	1
C11	16	2 Months	110	55	2
C12	20	2 Months	130	65	2
C13	25	2 Months	190	80	2

Abbreviations: DCC = Daycare Center; C = Center; D = Directors; NA = Not Available; No = Number.

**Table 3:** Table of quotes.

General Theme	Specific Topic	Quotes by Employees and Directors
Intention to support breastfeeding and perceived control	Encouragement of breastfeeding in DCC and how it affects the mother	“Our role [as DCC employees] would be to support the decision she [the mother] has already made. If she wants to breastfeed, we make her feel like a star.” (D1)
	Encouragement of breastfeeding in DCC and how it affects the mother	“We [DCC employees] do our best to help her [the mother] to ensure the baby gets the maximum amount of her milk until the last drop. After all, she [the mother] is working hard to produce that milk.” (E1)
Directors’ and employees’ knowledge and experience	Impact of DCC staff and director knowledge and/or experience on parents’ decision to enroll in DCC	“Parents trust us because they know we have experience.” (D7)
	Diversification and prioritization of training is necessary for the different employees	“Listen, the employees have so much on their plate. They are required to know so many things: the educational, behavioral and other aspects [of childcare]. I cannot add breastfeeding. They are also not required to have this [breastfeeding knowledge] as a skill set.” (D10)
	Impact of post-training certification on employee and director encouragement to learn	“The value of certification is beyond money. Especially these days [of economic hardship], that’s [education & skills] all we have.” (D2)
Attitude about breastfeeding in general and more specifically in the DCC	Benefits of breastfeeding	“Breastfeeding is the best for economic purposes, to save money otherwise used for powdered milk and pediatrician consultation costs, as well as emotional. I feel so close to my eldest child [who was breastfeed], which is different than with my other children [who were not breastfed]. The [connection through the] breast is different.” (D2)
	Practicality and feasibility of onsite breastfeeding in DCC	“The number of times the mom would have to drop by the DCC [to breastfeed] could be a limiting factor. It would be too much for the child. You can’t expose the child to phases of being so close [to the mother at the DCC], then complete separation [after she leaves]. There has to be [complete] weaning.” (D4)
	Acceptable child age for breastfeeding	“Until 9 months, and if the kid is OK with the separation [after breastfeeding] and will not cry due to the separation, I don’t have a problem in having the mother come [and breastfeed at the DCC]. We provide her a seat in the nursery, no camera, and she can breastfeed.” (D13) “In Lebanon, a small number of mothers breastfeed. Or you find the other extreme: people who breastfeed until 4 years of age. I honestly feel very weird about that. When the child has teeth, I feel it is abnormal to have the child breastfeed at that age.” (D13)
Political environment: policies and regulations at the macro- and micro-levels	Work policies for breastfeeding women	“If I have an employee who has just given birth and wants to breastfeed, she will be in and out of the nursery frequently. We try as much as we can to facilitate the process, but [eventually] the boss will ask, where is she? [It might work for] 1 or 2 months but then you start missing her at work, and you want her to be available. It is not my right to do so [to ask her to stop breastfeeding during work hours], but this is what happens.” (D13)
	Work policies for breastfeeding women	“I once read the [national] employment laws and learned that the breastfeeding mom should be given protected time to breastfeed. but I do not know if it is being implemented or not.” (E1)
	Policies and parents’ attitude toward breastfeeding	“I think if they [national policy makers] forbid breastfeeding, all women would breastfeed.” [in a sarcastic, ironic tone] (D13)
	Breastfeeding-friendly DCC certificate	“If the situation stays like this [economic crisis & pandemic], sanitation and [adequate] education will remain the most important [priorities]. A year ago, the situation was very different.” (D13)
	Breastfeeding-friendly DCC certificate	“This effort to participate in a breastfeeding certification program [in the future] will be done with all my heart. Maybe those notions [covered in a certification program] are missing in my DCC. Please teach them to me.” (D3)
	Breastfeeding-friendly DCC certificate	“Certainly, it is an advantage for the DCC as well [to be breastfeeding-certified]. Also, the woman who paid so much to get her educational degrees should not have to leave her work for childcare. If there is a DCC with these qualifications [to support breastfeeding], it is very good.” (E1)
Sociocultural environment: success stories, mother’s role…	Parents’ and mother’s role in breastfeeding continuation support	”I believe that the main role is for the parents.”(D1) “I: Do you try to change her mind? [talking about the employee convincing the mother to breastfeed].” E: Yes, but moms who do not have emotion [motivation] won’t do it.” (E1)
	Barriers for breastfeeding continuation for mothers	“It’s like new mothers feel like Cinderella. They are not always ready [to face breastfeeding challenges]. It is for them like a dream, so they do not expect breastfeeding.” (D5)
	Role of the mother in continuation of breastfeeding	“The mother herself is a very important factor in [continuing] breastfeeding. The environment around her has a 10-15% impact, no more. Based on my personal experience, I know that if I want to breastfeed, nothing will be in my way.” (D5)
	Success stories and motherhood impact on employees	“I have a student [child at DCC] aged 1 year and 8 months. His mother still sends [pumped] breastmilk for us to give him. I respect this very much… I think it’s still good for him and I encourage [her]. The boy is epileptic. I always handle this situation responsibly as I should, [as] I am a mom and I am an educator.” (E12)
	Attitude of director toward breastfeeding in DCC	“Mothers who do it [breastfeed] once or twice can always do it unless they do not want to. And in any DCC, I doubt that they [DCC team] would tell her not to breastfeed, or else she should have doubts about the DCC.” (D1)

## Data Availability

Data can be obtained from the first author upon reasonable request.

## Supplementary Materials

The following are available online at https://www.mdpi.com/article/10.3390/ijerph18126205/s1, Table S1: List of questions used in directors’ interviews, Table S2: List of questions used in employees’ interviews, Figure S1: Conceptual framework, Figure S2: Coding tree—employees, Figure S3: Coding tree—directors.

## References

[1] Bode L., Raman A.S., Murch S.H., Rollins N.C., Gordon J.I. (2020). Understanding the mother-breastmilk-infant “triad”. Science.

[2] Victora C.G., Bahl R., Barros A.J., França G.V., Horton S., Krasevec J., Murch S., Sankar M.J., Walker N., Rollins N.C. (2016). Breastfeeding in the 21st century: Epidemiology, mechanisms, and lifelong effect. Lancet.

[3] World Health Organization, UNICEF (2003). World Health Organization Global Strategy for Infant and Young Child Feeding.

[4] CDC (2015). Breastfeeding among U.S. Children Born 2002–2012.

[5] Batal M., Boulghaurjian C. (2005). Breastfeeding initiation and duration in Lebanon: Are the hospitals “mother friendly”?. J. Pediatric Nurs..

[6] Mattar L., Hobeika M., Zeidan R.K., Salameh P., Issa C., “Breastfeed for a Healthier Lebanon” Study Group (2019). Determinants of Exclusive and Mixed Breastfeeding Durations and Risk of Recurrent Illnesses in Toddlers Attending Day Care Programs Across Lebanon. J. Pediatric Nurs..

[7] Theurich M., Davanzo R., Busck-Rasmussen M., Díaz-Gómez N.M., Brennan C., Kylberg E., Bærug A., McHugh L., Weikert C., Abraham K. (2019). Breastfeeding Rates and Programs in Europe. A Survey of 11 National Breastfeeding Committees and Representatives. J. Pediatric Gastroenterol. Nutr..

[8] Hamade H., Chaaya M., Saliba M., Chaaban R., Osman H. (2013). Determinants of exclusive breastfeeding in an urban population of primiparas in Lebanon: A cross-sectional study. BMC Public Health.

[9] UNICEF Central Administration of Statistics, United Nations Children’s Fund Mulitple Indicator Cluster Survey—Lebanon 2009–2010. https://www.unicef.org/statistics/index_24302.html.

[10] Kuruvilla S., Bustreo F., Kuo T., Michra C., Taylor K., Forgstad H., Gupta G. (2016). The global strategy for women’s, children’s and adolescents’ health (2016–2030): A roadmap based on evidence and country experience. Bull. World Health Organ..

[11] Pérez-Escamilla R., Hromi-Fiedler A.J., Gubert M.B., Doucet K., Meyers S., Dos Santos Buccini G. (2018). Becoming Breastfeeding Friendly Index: Development and application for scaling-up breastfeeding programmes globally. Matern. Child Nutr..

[12] Rollins N.C., Bhandari N., Hajeebhoy N., Horton S., Lutter C.K., Martines J.C., Piwoz E.G., Richter L.M., Victora C.G. (2016). Why invest, and what it will take to improve breastfeeding practices?. Lancet.

[13] Pérez-Escamilla R., Curry L., Minhas D., Taylor L., Bradley E. (2012). Scaling up of breastfeeding promotion programs in low- and middle-income countries: The “breastfeeding gear” model. Adv. Nutr..

[14] BouDiab S., Werle C. (2017). What motivates women to breastfeed in Lebanon: An exploratory qualitative analysis. Appetite.

[15] Nabulsi M. (2011). Why are breastfeeding rates low in Lebanon? a qualitative study. BMC Pediatrics.

[16] Batal M., Boulghourjian C., Abdallah A., Afifi R. (2006). Breast-feeding and feeding practices of infants in a developing country: A national survey in Lebanon. Public Health Nutr..

[17] Akik C. (2014). Breastfeeding in Lebanon: Barriers and Policy Dynamics. Ph.D. Thesis.

[18] Economou M., Kolokotroni O., Paphiti-Demetriou I., Kouta C., Lambrinou E., Hadjigeorgiou E., Hadjiona V., Tryfonos F., Philippou E., Middleton N. (2018). Prevalence of breast-feeding and exclusive breast-feeding at 48 h after birth and up to the sixth month in Cyprus: The BrEaST start in life project. Public Health Nutr..

[19] Vieira T., Vieira G., Giugliani E., Mendes C., Martins C., Silva L. (2010). Determinants of breastfeeding initiation within the first hour of life in a brazilian population: Cross-sectional study. BMC Public Health.

[20] Bueno-Gutierrez D., Chantry C. (2015). Determine breastfeeding obstacles in a low-income population in Tijuana, Mexico: Healthcare services. Breastfeed. Med..

[21] Navarro-Rosenblatt D., Garmendia M. (2018). Maternity Leave and its impact on breastfeeding: A review of the literature. Breastfeed. Med..

[22] Munn A., Newman S., Mueller M., Taylor S. (2016). The impact in the United States of the baby-friendly hospital initiative on early infant health and breastfeeding outcomes. Breastfeed. Med..

[23] Singletary N., Chetwynd E., Goodell S.L., Fogleman A. (2017). Stakeholder views of breastfeeding education in schools: A systematic mixed studies review of the literature. Int. Breastfeed. J..

[24] Moukarzel S., Abou Jaoudeh M., Farhat A., Saade M., Mamas C., Daly A.J. (2020). Exploring the latitude of attitude: Intentions to breastfeed among adolescents in Lebanese schools. Matern. Child Nutr..

[25] Moukarzel S., Mamas C., Warstadt M.F., Bode L., Farhat A., Abi Abboud A., Daly A.J. (2018). A case study on breastfeeding education in Lebanon’s public medical school: Exploring the potential role of social networks in medical education. Med. Educ. Online.

[26] Redford J., Desrochers D., Hoyer K.M. (2017). The Years before School: Children’s Nonparental Care Arrangements from 2001 to 2012.

[27] Lundquist A., McBride B.A., Donovan S.M., Kieffer A. (2019). An Exploratory Look at the Role of Childcare Providers as a Support and Resource for Breastfeeding Mothers. Breastfeed. Med. Off. J. Acad. Breastfeed. Med..

[28] Marhefka S.L., Sharma V., Schafer E.J., Turner D., Falope O., Louis-Jacques A., Wachira M.M., Livingston T., Roig-Romero R.M. (2019). ‘Why do we need a policy?’ Administrators’ perceptions on breast-feeding-friendly childcare. Public Health Nutr..

[29] Saade N., Barbour B., Salameh P. (2010). Maternity Leave and experience of working mothers in Lebanon. East Mediterr. Health J..

[30] Chami M., Mikhael M. (2016). The Saga of the Lebanese Healthcare Sector: Reforms on the Run Amid Persistent Challenges.

[31] Nursery Registration Process. https://www.moph.gov.lb/en/DynamicPages/index/3/4021/nurseries.

[32] Infant Formula (from 0 to 1 year). https://www.moph.gov.lb/en/Pages/3/25171/infant-formulas-from-0-to-1-year-.

[33] WHO. https://www.who.int/nutrition/topics/infantfeeding/en/.

[34] Clark A., Anderson J., Adams E., Baker S. (2008). Assessing the knowledge, attitudes, behaviors and training needs related to infant feeding, specifically breastfeeding, of child care providers. Matern. Child Health J..

[35] Javanparast S., Sweet L., Newman L., McIntyre E. (2013). A survey of child care centers about breastfeeding support in Adelaide, South Australia. J. Hum. Lact. Off. J. Int. Lact. Consult. Assoc..

[36] Mohd Suan M.A., Ayob A., Rodzali M. (2017). Childcare workers’ experiences of supporting exclusive breastfeeding in Kuala Muda District, Malaysia: A qualitative study. Int. Breastfeed. J..

[37] Lucas A., McMahon P., Asling M., Knobloch A., Kosh E., Sims K. (2013). Assessing child care providers’ knowledge and attitudes regarding support of breastfeeding in a region with low breastfeeding prevalence. J. Hum. Lact..

[38] Kremers S.P.J., de Bruijn G.-J., Visscher T.L.S., van Mechelen W., de Vries N.K., Brug J. (2006). Environmental influences on energy balance-related behaviors: A dual-process view. Int. J. Behav. Nutr. Phys. Act..

[39] Hector D., King L., Webb K., Heywood P. (2005). Factors affecting breastfeeding practices: Applying a conceptual framework. New South Wales Public Health Bull..

[40] Manhire K.M., Horrocks G., Tangiora A. (2012). Breastfeeding knowledge and education needs of early childhood centre staff. Community Pract. J. Community Pract. Health Visit. Assoc..

[41] Javanparast S., Newman L., Sweet L., McIntyre E. (2012). Analysis of breastfeeding policies and practices in childcare centres in Adelaide, South Australia. Matern. Child Health J..

[42] Zapana P.M., Oliveira M.S., Taddei J.A. (2010). Factors determining the breastfeeding in children attending public and not-for-profit daycare centers in São Paulo, Brazil. Arch. Lat. Nutr..

[43] Batan M., Li R., Scanlon K. (2013). Association of child care providers breastfeeding support with breastfeeding duration at 6 months. Matern. Child Health J..

[44] Garth E., Messer A.L., Spatz D.L. (2016). Child Care Centers’ Role in Support of Breastfeeding Families. MCN Am. J. Matern. Child Nurs..

[45] Akik C., Ghattas H., Filteau S., Knai C. (2017). Barriers to breastfeeding in Lebanon: A policy analysis. J. Public Health Policy.

[46] Souza J.P., Prudente A.M., Silva D.A., Pereira L.A., Reinaldi A.M. (2013). Evaluation of employees in public day care centers knowledge about breastfeeding and complementary feeding. Rev. Paul. Pediatr..

