# Supplementary material for: Factors Related to Breastfeeding Support in Lebanese Daycare Centers: A Qualitative Study among Daycare Directors and Employees
Source: Int J Environ Res Public Health. 2021 Jun 8;18(12):6205. doi: 10.3390/ijerph18126205 (PMC8228840; doi:10.3390/ijerph18126205)

Figure S2 – Coding tree for employees' interviews

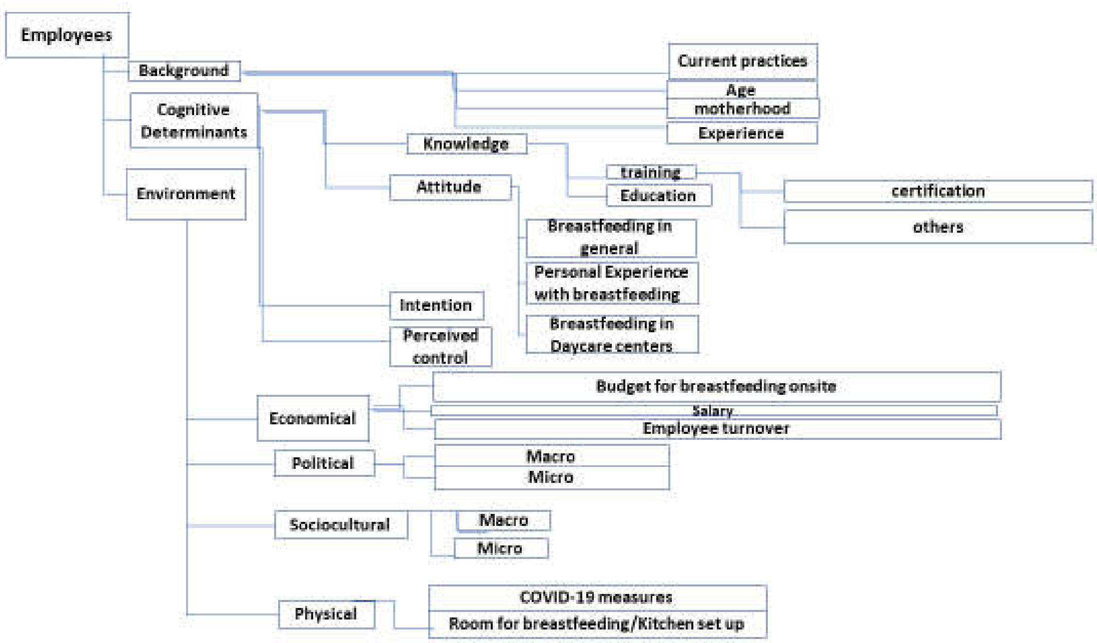

Figure S3- Coding tree for directors' interviews (BF=breastfeeding)

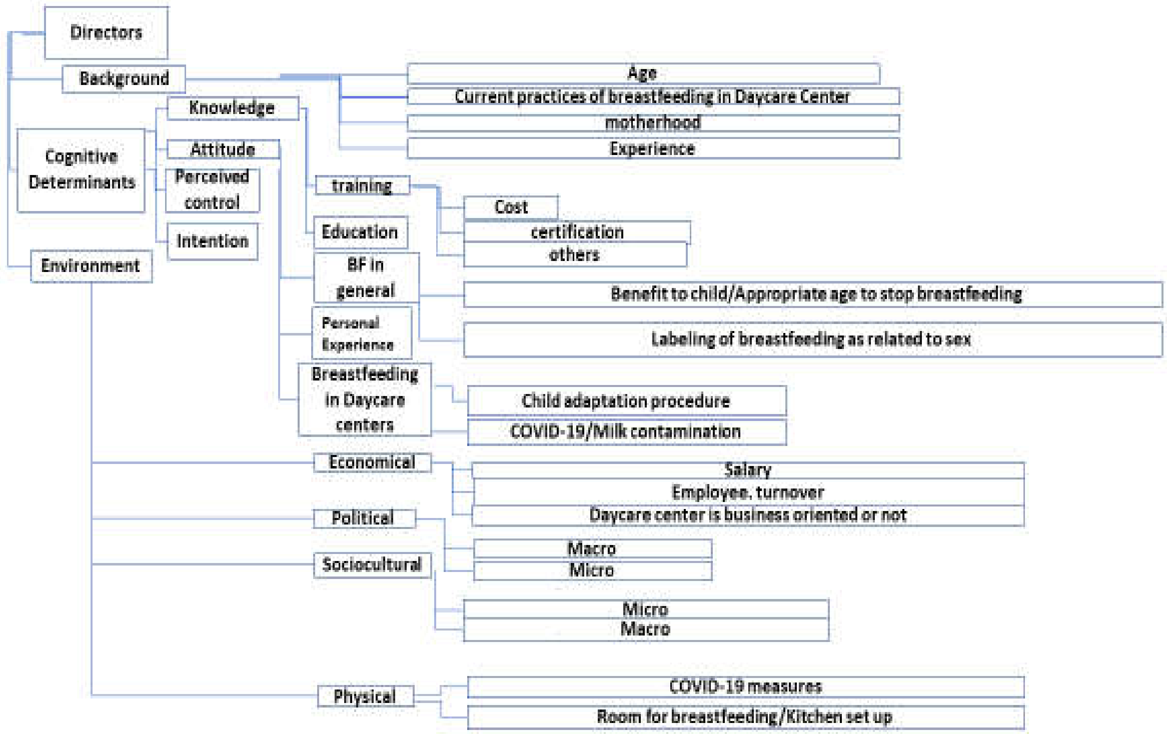

Supplement: Supplementary file 1 [file ijerph-18-06205-s001.zip › Supplementary Material-figures S2 and S3-Coding trees.pdf]
