# Supplementary material for: Factors Related to Breastfeeding Support in Lebanese Daycare Centers: A Qualitative Study among Daycare Directors and Employees
Source: Int J Environ Res Public Health. 2021 Jun 8;18(12):6205. doi: 10.3390/ijerph18126205 (PMC8228840; doi:10.3390/ijerph18126205)
Supplement: Supplementary file 1 [file ijerph-18-06205-s001.zip › Supplementary Material-Table S1 and S2-Questions for directors and employees.pdf]

Table S1: Questions for directors

**Personal information:**							
Age	___	Years					
Gender	☐	Female	☐	Male			
Marital status	☐	Married	☐	Not married			
Do you have children	☐	Yes	☐	No (skip to question 6)			
If yes, specify how many	___						
Were your children breastfed?	☐	Yes	☐	No			
If yes, please specify for how many months on average:	___						
Highest degree	☐	Baccalaureate	☐	Bachelor	☐	Masters or higher	
For how many years have you been working in your profession?	___						
For how many years have you been working in this specific day care center?	___						
**Daycare center characteristics:**<br>-How long has this nursery been operating?<br>-How many babies are there in the daycare center?<br>-What is the age range accepted?<br>-How many employees work in this daycare center?<br>-What is their job description?							
**Breastfeeding**							
-What are your thoughts about breastfeeding in general? And in the daycare center?<br>-Do you think it is important?							
**Director's personal home environment**							

How does your home environment affect your readiness to support breastfeeding? (Your own parents, your religion, the environment you live in, region, building etc…<br>-Do you have any personal or family experience that might affect your support of breastfeeding?
**Breastfeeding in the daycare center**
-What is your exact role in the daycare center? (details if you please)<br>-How are the different tasks generally divided in the daycare center?<br>-Can you elaborate on who is responsible of feeding the infants/youngest children?<br>-What about the kitchen premises? Any specific person responsible?<br><br>-How many children attend the daycare center you work in? How many are breastfed?<br>-If the children are taking breastmilk, which form is the most common? (onsite breastfeeding, bringing pumped milk from home or onsite pumping)<br>-Have you ever had a mother breastfeed or pump on premise? How was the experience? What were the different barriers to that?<br><br>-In the Lebanese daycare center set up in general, what are the barriers and supports of breastfeeding that you can think of? And do they apply to your specific center?<br>*Physical* (availability of space, fridge, work area) (electricity, formula availability in market)<br><br>*Political* (daycare center policies or national policy, policies on education of employee, implementation of those policies in the daycare center, prioritization of breastfeeding from a national stand point, monitoring by the government on breastfeeding practices in daycare centers)<br><br>*Economical* (time consuming and pressure on the employees, costly to set up this system in the daycare center, costs of formula)<br><br>*Sociocultural* (mother's readiness and return to work, taboo to breastfeed in public, the norm is to breastfeed until starting daycare, breastmilk is not sufficient, breastfeeding is not the norm in the family ,parents decision, general daycare center practices)
**Communication with parents**
-Upon parents' arrival at the daycare, do you discuss with them the type of milk they want to give their child and the way they want to give it?<br>- Do you offer your opinion? Do you try to convince them to breastfeed or not given the set up you have?<br>-what do parents usually want?<br><br>- Are the parents in your daycare center provided with an information pack when they first enroll their child? Is information on breastfeeding support part of the package?<br>-Do you have breastfeeding related printed material available in your daycare center? (Like posters, leaflets or children's books tackling breastfeeding)<br><br>-**Regarding oral communication**:<br>-Do you promote breastfeeding continuation at child's enrollment and after?<br>-How do you communicate with the parents along the way?

-Is information about breastfeeding also discussed orally with parents like daily attendance and activities?

**If you do have the written and/or oral communication with parents, how do you see this affecting their initial readiness/willingness to continue breastfeeding in the daycare center?

The Mother

-Do you encourage mothers to come breastfeed in the daycare center?
-How do you see the conditions mothers have at their work affecting their attitude towards continuation of breastfeeding?
-Do you think the neighborhood the mother is living in (usually close to the daycare center) with all its traditions/customs affects the mothers' readiness to breastfeed?
-Are mothers who live in specific districts more or less prone to be ready to breastfeed?

-Do you think the daycare center's management, policies, employees' attitude, readiness to support affect the mothers' readiness to continue breastfeeding?
-Do you think that encouraging mothers and providing private space for them to breastfeed would help in increasing the occurrence and continuation of breastfeeding?

Physical set up

-Do you have fridges, equipped kitchens for breastmilk handling (usage of convenient containers for reheating)?
-Bottles to reheat milk
-Do you have a specific private, closed room to practice pumping/breastfeeding?
-How do you see this affecting the employees' attitude, ability, readiness and perceived success to support breastfeeding in the daycare center?

training

-Do you have any breastfeeding training, prepared and given to your staff?
-Have you had breastfeeding and breastmilk handling trainings offered by Non-Governmental Organizations or by the government?
-Do you have breastfeeding consultants' visits to the daycare? Do they have contact with your employees or parents?
-Do you think these trainings would improve the breastfeeding rates in the Daycare center?
-Do they affect the employees' practice/attitude/intention in any way?

-Do you have breastfeeding practices webinars or other online resources related to breastfeeding available to employees?
-Do you encourage them to access them?
-What about technology in general? Do the employees consider it as a resource of choice for trainings?
-Do you think this type of learning affects the breastfeeding rates in your Daycare center?

-do you provide any **certification** for your employees if they do have trainings of the kind?
-Do you think certification would increase their interest and affect their involvement in the support of breastfeeding?

**Organizational factors**
- How do you see the staff's attitude, intention, self-efficacy and knowledge affecting breastfeeding rates in the daycare center?<br>-How about their education and background?<br><br><br>-Do management practices, communication with employees, job stability, turnover rate affect the breastfeeding support from the employees' point of view?<br><br>-How does the daycare center environment affect your intention and capacity to support breastfeeding in the daycare center? (In terms of colleagues' attitudes, intentions, culture, religion…)
**Policies**
-Do you have knowledge of national breastfeeding policies for daycare centers?<br>-Do you have an infant feeding policy (possibly written) specific to your daycare center?<br>-Is this policy communicated to both parents AND staff?<br>-Is policy review practiced?<br>-Do these policies affect your attitude, capacity and intention to support breastfeeding in the daycare center?<br>-During/after COVID-19 pandemic, do you have any specific regulations regarding the presence of parents (especially mothers) on premise and breastfeeding on premise?<br>-Any implementation of new recommendations or regulations in the light of safety measures taken for COVID-19?
**Becoming a breastfeeding friendly daycare center**
-How important is it for you to become a breastfeeding friendly daycare center?<br>-Do you think this would increase parents trust and willingness to enroll their children in your daycare center?<br>-Which barriers do you anticipate? And what facilitates change?<br>-Do you think you can do it?
**Final comments**
Any additional comments?

Table S2- Questions for Employees

Personal information:

	Age	___	Years				
[illegible]	Gender	☐	Female	☐	Male		
[illegible]	Marital status	☐	Married	☐	Not married		
[illegible]	Do you have children?	☐	Yes	☐	No (skip to question 6).		
	If yes, specify how many:	___					
[illegible]	Were your children breastfed?	☐	Yes	☐	No		
	If yes, specify for how many months on average:	___					
[illegible]	Highest degree	☐	Baccalaureate	☐	Bachelor	☐	Masters or higher
[illegible]	Specify your work	☐	Nurse	☐	Teacher/ teacher aid	☐	Helper
[illegible]	For how many years have you been working in your profession generally?	___					
[illegible]	For how many years have you been working in this daycare?	___					
	Do you currently work with infants aged 3 months up to 2 years of age?	☐	Yes	☐	No		

Breastfeeding

-What are your thoughts about breastfeeding in general?
-Do you think it is important?

Breastfeeding in the daycare center

-What is your exact role in the daycare center? (details if you please)
-How are the different tasks divided among the employees?
-Can you elaborate on who is responsible of feeding the infants/younger children?
-What about the kitchen premises? Any specific employee responsible?

-How many children attend the daycare center you work in? How many are breastfed?
-If the children are taking breastmilk, which form is the most common? (onsite breastfeeding, bringing pumped milk from home or onsite pumping)
-In practice, have you ever had a mother breastfeed/pump on premise or pump at home and bring her milk with her? How was the experience? What were the different barriers to that?

-In the Lebanese daycare center set up in general, what are the barriers and supports of breastfeeding that you can think of? (here I ask about the two separately)
Barriers-Supports:
Physical (availability of space, fridge, work area) (electricity, formula availability in market)

Political (daycare center policies or national policy, education of employee, implementation of those policies in the daycare center, prioritization of breastfeeding from a national stand point)

Economical (time consuming and too much pressure on the employees, costly to set up this system in the daycare center) (monitoring by the government on breastfeeding practices in daycare centers)

Sociocultural (mother's readiness and return to work, taboo to breastfeed in public, the norm is to breastfeed until starting daycare, breastmilk is not sufficient, breastfeeding is not the norm in the family, parents decision, general daycare center practices)

Role of the employee

-How important is the role that a daycare employee plays in regard to implementation of breastfeeding in the daycare center?

-***Attitude:*** What do you think about the continuation of breastfeeding in the daycare center through expressed milk or breastfeeding on site?
-***Intention and Self-efficacy:*** Is this an activity that you want and most importantly feel able to carry on?
-Do you feel you are in control of your behavior of the support you can give to mothers? (your decision is only your own)

- What mostly affects (barriers/supports)your ability to support mothers in breastfeeding?

-Does your home environment affect your readiness to support breastfeeding? (Your own parents, your religion, the environment you live in, region, building etc…<br>-Do you have any personal or family experience that might affect your support of breastfeeding?<br><br>-Does media like TV, newspapers etc…affect your support of breastfeeding?
**Mother/Parents**
-How do you communicate with parents (written, oral) regarding attendance and other issues (like breastfeeding)?<br>-Do you verbally promote breastfeeding continuation? (When you first meet the parents and in later times)<br><br>-How do the mothers' or parents' attitude and their readiness to breastfeed affect their continuation of breastfeeding in the daycare center?<br>-Does the latter have an impact on your readiness, intention and perceived self-efficacy to support breastfeeding continuation?<br>-Also, is it the other way round? (does your readiness affect the parents'?)<br><br>-When parents have newly enrolled their child in the daycare center, do you discuss with them the type of milk they want to give him/her and the way they want to give it?<br>-Do you discuss your own opinions about breastfeeding with the parents or do you abide by their wishes<br><br>-Does the mother's work schedule, maternity leave length or work location affect her readiness to breastfeed?<br><br>-Do you happen to have any success stories that increased your confidence in supporting breastfeeding?
**Facilities/Physical set up**
-Does your facility have all necessary equipment/space to provide breastfeeding assistance to mothers?<br>(Breast pumps, private closed room, kitchen equipment and counters…).<br><br>-How do you see the availability of these resources affecting your attitude, ability, readiness and perceived success to support breastfeeding in the daycare center?
**Organizational factors**
-What about the director's?<br>attitude, daycare management strategy, communication of policies, communication skills in general, relation with employees<br>-Do you think it affects the breastfeeding support in the daycare center?<br>-How does the team dynamics affect the willingness of employees to accept assisting mothers in the breastfeeding process? (Employee turnover, colleagues attitudes, job stability, updating new employees about work details…)<br>-Do you think the number and/or job description of employees affects the ability to give breastfeeding support to mother?

-How does the daycare center environment affect your intention and capacity to support breastfeeding in the daycare center? (In terms of culture, religion…)
**Trainings**
- Did you receive any breastfeeding support training in the daycare center or during your university/technical education? If yes, where? (check if through university or through continuing education (conferences, workshops etc) or in the daycare center.<br>-Do you have access to webinars or other online resources related to breastfeeding?<br>-How do you see this affecting your perspective on breastfeeding support in the daycare center?<br>-After completing these trainings, do you have the knowledge and skills to practice breastfeeding support in the daycare center?<br><br>-What kind of information and training do you think are needed to be able to assist the parents with their child's feeding?<br>(Basic info about breastfeeding benefits for the mom and baby, reheating milk, storage of breastmilk, food safety practices etc… or more developed, or tackling certain sides of the topic)<br><br>-Would having a reward or a certification be a driving incentive to complete such trainings?
**Policies**
-Are you also aware of a national policy about breastfeeding?<br>- Are you aware of a written breastfeeding policy that is implemented in the daycare center you work in?<br>-how is this policy communicated to you?<br><br>-Does the policy make sense to you?<br>-Does this policy encourage you to support breastfeeding and how :<br>They affect your intention to perform breastfeeding support.<br>They increase your capacity of supporting breastfeeding.<br>They affect your opinion about breastfeeding.
**Additional comments**
Do you have any comments to add?
